# Abstracts of the 10th DACH+ Conference on Energy Informatics: Virtual,13-17 September 2021 https://energy-informatics2021.org/

**DOI:** 10.1186/s42162-021-00184-2

**Published:** 2021-09-13

## INTRODUCTION

### P1 Welcome message from the organizers

#### Anke Weidlich^1^, Gunther Gust^2^, Mirko Schäfer^1^

##### ^1^INATECH, University of Freiburg, Emmy-Noether-Str. 2, 79110, Freiburg, Germany; ^2^Chair for Information Systems Research, University of Freiburg, Rempartstr. 16, 79098, Freiburg, Germany

#### **Correspondence:** Anke Weidlich (anke.weidlich@inatech.uni-freiburg.de)

Dear readers,

In this supplement of the proceedings of the 10th DACH+ Conference on Energy Informatics 2021, we present the extended poster and demo abstracts, including two contributions from the co-located Energy Informatics Doctoral Workshop.

Sincerely,

Anke Weidlich (General Chair)

Gunther Gust (Poster Chair)

Mirko Schäfer (Publication Chair)

Acknowledgements

Not applicable.

Funding

Publication funding was provided by the German Federal Ministry for Economic Affairs and Energy.

Availability of data and materials

Not applicable.

Author’s contributions

The authors read and approved the final manuscript.

Competing interests

The authors declare that they have no competing interests.

## DEMO ABSTRACT

### P2 A Platform to Assess the Trust in Power System Components, Data, and Services

#### Michael Brand, Felipe Castro, Batoul Hage Hassan, Carsten Krüger, Torben Logemann, Björn Siemers, Dennis Weller, Torge Wolff, Sebastian Lehnhoff

##### OFFIS – Institute for Information, Technology, Energy Division, Escherweg 2, Oldenburg, Germany

###### **Correspondence:** Michael Brand (michael.brand@offis.de)

Summary

Modern energy systems are cyber-physical energy systems (CPES) with increased reliance on information and communication technology (ICT). With the ”Smart Grid Cyber-Resilence Laboratory”, a laboratory is built up to enable and foster the research on the influence of data integrity losses on the functionality of a CPES. This paper describes a demonstration of how the situational awareness of the ICT system can contribute to reliable power system operation. Monitoring systems are deployed, and their data are fed into a big-data time-series database. The information is used to assess the trust in power system measurements and state variables. In the demonstration, the operator can keep the system stable with help of hints about a manipulation in terms of untrustworthy state variables.

Introduction

A cyber-physical energy system (CPES) needs to ensure a base functionality (i.e., reliable power supply) even in cases of temporary losses of the integrity or trustworthiness of the communication. To enable and foster the research on this topic, the ”Smart Grid Cyber-Resilence Laboratory”^1^ (CybResLab) has recently been set up. The CybResLab aims at augmenting power system process data with information about the integrity of the data sources and information and communication technology (ICT) infrastructure. This distinguishes the CybResLab from other laboratories or platforms, which in most cases focus on either simulation of CPES or vulnerability assessment (cf., for example, the EnergyShield project [1] or the Energy Lab 2.0 [2]). In the CybResLab, components for real-time co-simulation, monitoring, and controlling of a CPES are integrated into a functional platform. Available components are, for example, state-of-the-art virtual/physical remote terminal units (v/RTUs), intrusion detection systems (IDSs), ICT health monitoring systems, and a common big data platform.

This paper demonstrates of how the operation of CPES can benefit from the integration of the trustworthiness of data sources and the ICT infrastructure. Trust is defined to be ”a subjective, context-dependent, and multivariate sense about an entity with respect to its functional correctness, safety, security, reliability, credibility, and usability” [3]. For example, information from an intrusion detection system can be used to derive a value for the security facet and information about resource usage of devices from an ICT health monitoring system for the functional correctness.

A setup is configured for this purpose, which contains a co-simulation of a CPES, monitoring of the ICT devices, and storing all power system measurements and monitoring data in a big data platform. The integrity and trustworthiness of power system measurements and state variables are calculated from that information and considered in the operation of the system. The setup is an extension of a former publication on the theoretical concept [4].

Platform

The components of the platform can be categorized into three categories, namely simulation, monitoring, and application tools, as shown in Figure 1. The power system is a modified version of the CIGRE medium voltage distribution grid^2^ with 12 instead of 15 busbars. A transformer is connected to a residential area with a high installed capacity of Distributed Energy Resources (DER), namely photovoltaic and wind farms. The real-time simulation of the power system is done in OPAL-RT, which is connected to vRTUs, one per busbar, developed at OFFIS [5]. The communication network consisting of all connections between the simulation, monitoring, and application components is simulated with EXata, a real-time communication emulator. The communication network in the demo is a meshed core network with wired connections between all components. The network consists of four core routers, control center components, and one edge router per vRTU.

Checkmk is used as an IT monitoring tool to monitor the behavior of IT and OT devices within the network. Each vRTU in the demo is equipped with a software agent providing status information like CPU usage, memory usage, data flow per second, and process information. In addition, an IDS (suricata) is actively inspecting the network in scope to detect malicious behavior such as attacks on the network or harmful payload. It is installed on a separate server with a network interface to the inspected network running in promiscuous mode.

A central data platform is used to store, enrich, and analyze the data. The platform ingests all data from all devices via redis and logstash and stores it in an elasticsearch database. Services can read the data from the database and store new data in the database. In the demo, two services make use of the data platform. First, an anomaly sensitive state estimation [3, 6], estimates the system state and annotates each state variable with multi-faceted trust values. A trust estimation for each measurement based on the information from the IT monitoring system and the IDS precedes the state estimation. Second, the local outlier factor algorithm [7] is used as a model for host-specific anomaly detection. It has the goal of detecting anomalies (such as cyber attacks) on the vRTUs in the demo. The last component in the setup is a graphical user interface realized as a web application with Grafana. In Grafana, all relevant information about the current state of the system is visualized as will be explained in more detail in the following section.

Use Case

The key idea behind the demo is to show that the trustworthiness of measurements can play a major role in preventing undesired (manual or autonomous) reactions of the power system. In particular, the demo consists of a coordinated false data injection attack (FDIA), in which the control of four vRTUs is achieved by an intruder. The intruder gains access to the vRTUs by using multi-staged exploits on a vulnerable FTP server. After gaining access, the intruder is able to intercept the measurements received from the power system simulator and inject manipulated measurements. The objective of the coordinated FIDA is to stay below the bad data detection threshold with the manipulations and to mimic an undervoltage situation and trigger a tap change by the transformer’s tap controller. Because of the manipulated measurements, that tap change then in reality causes an overvoltage situation. The operator can avoid the tap change and keep the system stable with help of an automatic detection that state variables are untrustworthy, derived from anomalies detected in the ICT health monitoring system and the IDS. For example, alerts from the IDS give hints for an illegal access to the vRTUs.

Via the graphical user interface, the operator is able to see the operating state of the power system and the main characteristics of the ICT system that sends all the information retrieved in the field to the control room (cf. Figure 2). In addition, the operator can also see the results of the anomaly sensitive state estimation with detailed information about the trustworthiness of the state variables (cf. Figure 3).

The value of each trust facet is visualised in a spider diagram. Several aspects can contribute to a single facet. Therefore, single trust values that contributed to the aggregated trust value are shown in the tables on the right.

Conclusion

This paper described a demonstrator that shows the benefit of using information about the trustworthiness of data for system operators to control a CPES. In the demonstration, a CPES is co-simulated in real-time, vRTUs are monitored by an ICT health monitoring system, and the ICT network by an IDS. With the information from the monitoring systems, the trustworthiness of measurements and state variable is estimated and integrated into a graphical user interface. A false data injection attack suggests performing a tap change. Reduced trust values let the operator not perform the tap change. Therefore, the system remains stable.

Funding

The research project Smart Grid Cyber-Resilience Lab is funded by the Federal Ministry for Economic Affairs and Energy under the agreement no. 0350008.

Availability of data and materials

There is no data or material publicity available yet.

Author’s contributions

All authors contributed equally to the development of the demonstrator.

Competing interests

The authors declare that they have no competing interests.

References

1. Georgiadou, A., Mouzakitis, S., Kanaris, B., Askounis, D.: A cyber-security culture framework for assessing organization readiness. Journal of Computer Information Systems 0(0), 1–11 (2020)

2. Düpmeier, C., Stucky, K.-U., Mikut, R., Hagenmeyer, V.: A concept for the control, monitoring and visualization center in energy lab 2.0, 83–94 (2015)

3. Brand, M., Babazadeh, D., Krüger, C., Siemers, B., Lehnhoff, S.: Trust assessment of power system states. Energy Informatics 3(1), 1–11 (2020)

4. Brand, M., Ansari, S., Castro, F., Chakra, R., Hassan, B.H., Krüger, C., Babazadeh, D., Lehnhof, S.: A framework for the integration of ict-relevant data in power system applications. In: 2019 IEEE Milan PowerTech, pp. 1–6 (2019). IEEE

5. Ansari, S., Castro, F., Weller, D., Babazadeh, D., Lehnhoff, S.: Towards virtualization of operational technology to enable large-scale system testing. In: IEEE EUROCON 2019-18th International Conference on Smart Technologies, pp. 1–5 (2019). IEEE

6. Brand, M., Babazadeh, D., Lehnhoff, S.: Trust in Power System State Variables Based on Trust in Measurements. in press

7. Breunig, M.M., Kriegel, H.-P., Ng, R.T., Sander, J.: Lof: Identifying density-based local outliers, 93–104 (2000)

**Fig. 1 (abstract P2).**
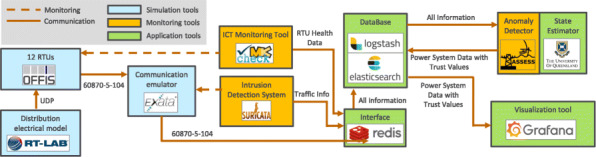
Real-time co-simulation platform for a trust assessment in a CPES

**Fig. 2 (abstract P2).**
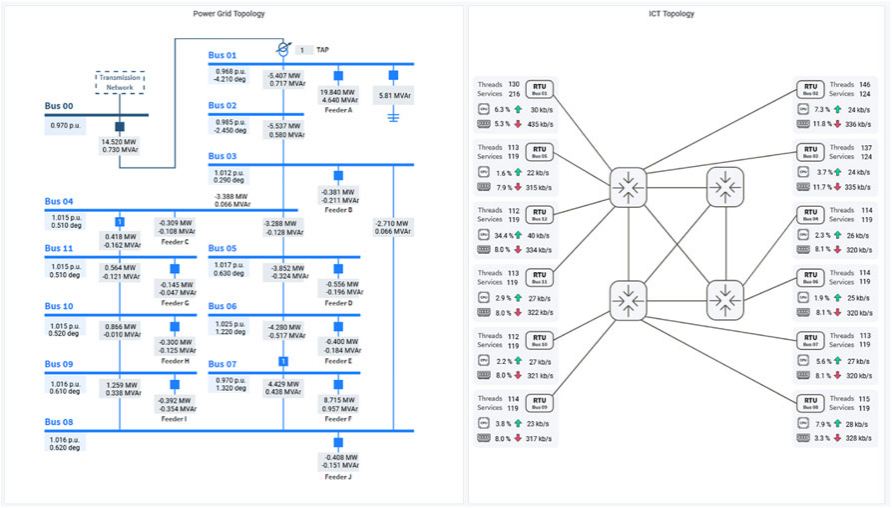
Combined visualization of the topologies, enriched with live data

**Fig. 3 (abstract P2).**
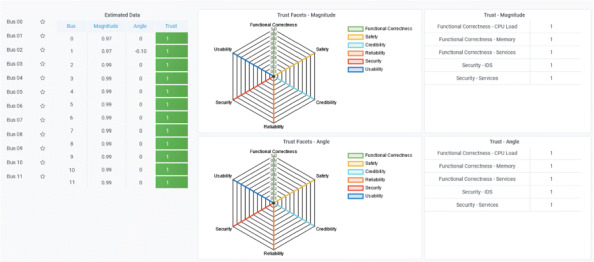
Visualisation of the state variables, enriched with multi-faceted trust values

## POSTER ABSTRACT

### P3 An Optimization Approach for Designing an Energy System Based on Renewable Energy Sources for an Austrian Farm

#### Lukas Gnam^1^, Bertram Gschwandtner^1^, Ina V Tomaschitz^2^, Christian Pfeiffer^2^

##### ^1^ Fachhochschule Burgenl and GmbH, Department of Energy and, Environment, Steinamangerstraße, 21, 7423, Pinkafeld, AT

###### **Correspondence:** Lukas Gnam (lukas.gnam@fh-burgenland.at)

Summary

Renewable energy sources are key to overcoming the challenge of transforming the existing centralized energy systems into sustainable, decentralized, and green systems. However, these energy sources require a critical and limited resource: space. In Austria, on the one hand, about two percent of the final energy consumption is used in agriculture. On the other hand, farms offer optimal conditions for installing several different renewable energy sources as there are often large structures and buildings offering huge spaces for potential mounting of photovoltaic or solar thermal systems. Therefore, this study focuses on the formulation of a mixed-integer linear optimization model in order to derive optimal sizes of renewable energy systems for electricity and heat production. The model is evaluated utilizing different objectives, i.e., an economically feasible inclusion of RESs and achieving autarky without any grid connection. Keywords: mixed-integer linear programming; renewable energy sources; farm

Introduction

Climate change poses enormous challenges regarding present and future energy systems as these are a major pollutant of green house gas emissions [1]. Hence, it is necessary to include more and more renewable energy sources (RESs), like wind and solar power, into the existing energy systems. However, RESs require a critical and limited resource: space. Therefore, one option is to utilize already used space for the installation of these RESs. In Austria, about two percent of the final energy consumption is used in agriculture [2], offering enormous potential for directly producing and consuming renewable energy on site. Additionally, farms offer optimal conditions for installing several different RESs as there are often large structures and buildings offering huge spaces for potential mounting of photovoltaic (PV) or solar thermal (ST) systems.

Here, mathematical modelling and optimization play a vital role in exploring the pros and cons of different technical solutions and investment decisions [3]. One prominent mathematical method is the Mixed-Integer Linear Programming (MILP) approach [4], where the model formulation is based solely on linear functions. The MILP modeling of energy systems is a prominent field of research. For example, various researchers investigated multi-energy systems [5, 6, 7]. However, regarding the agricultural sector most research is focused on a holistic modeling and analysis of food supply chains [8] or the optimal use of resources in rural areas [9].

Therefore, this study focuses on the formulation of a MILP model of the energy system of an Austrian farm in order to derive optimal sizes of RES-based electricity and heat plants as well as computing schedules for the different components to achieve minimum costs. The MILP model is evaluated utilizing different objectives, i.e., including RESs and achieving autarky.

Optimization Model

The optimization model presented in this work is based on a typical Austrian farm of about 45 ha and an estimated roof size of 500 m^2^. The investigated system consists of the following different consumers and producers. Due to the available roof space, PV and ST systems are the most prominent choices for providing electrical and thermal energy [5, 10] and are thus integrated as investment options in the conducted study. Wind turbines are another option for generating green electricity and, although, several studies concluded that they are hardly economically feasible [11], we included these technologies into our investigations. This allows for an investigation of potential policy or subsidy changes in order to increase the attractiveness of wind turbines. Another source for heat energy production are wooden-fueled heating plants. For farms it is also an interesting option to install plant-based combined heat and power plants (CHPs), as the required biomass for producing the plantbased fuel can be grown on the farm. To enable load shifting both electrical storage system (ESSs) and thermal storages are included in the conducted computational study. On the consumption side of the farm’s energy system the electrical demand of the household as well as its heating and domestic hot water demand are included in the MILP model. Both data are taken from from the APCS Power Clearing and Settlement AG [12] and the AGCS Gas Clearing and Settlement AG [13]. Furthermore, the flexible utilization of an electricity-based plant oil pressed is evaluated as decision variable. As meat production on farms requires a cool room, the electricity consumed by such an installation is also taken into account as static demand profile. To ensure mobility for the farm’s inhabitants the demand of an electrical vehicle represents another electrical consumer in the system.

Results and Discussion

In this section the obtained results for two different scenarios are discussed. Scenario 1 covers an economically feasible integration of RESs and Scenario 2 evaluates how full autarky of the farm’s energy system is possible. Regarding the electrical energy of the system the degree of self sufficiency (DSS) $DSS=\frac{E_{SC}}{E_V}$, the self consumption rate (SCR) $SCR=\underset{E_E}{E_{sc}+E}$ ^ESS^ , and the degree of autonomy (DA) $DA=\frac{E_E}{E\kern0.48em E\kern0.72em }$ *V* + ESS are evaluated. The simulation data obtained show that in Scenario 1 an investment into a 7 kWp PV system achieves an SCR of 60.8 %. Although various different RESs are available only the PV system is an economically feasible solution with respect to the constraints and assumed cost data. Subsequently, about 93 % of the available roof space remain unused for energy production.

In Scenario 2 the optimization results yield a 10 kWp PV system as optimal solution for using the available roof space coupled with a 21 kWh ESS. Additionally, in this scenario the investment into a CHP with 3 kW_el_ and a thermal storage of 7 m^3^ are recommended. In contrast to Scenario 1, the assumed autarky leads to the highest annual costs of all investigated scenarios. This is mostly due to the fact that no surplus energy can be sold. Furthermore, the SCR is about 87 % and the DA is more than 114 %. The SCR below 100 % can be explained by losses in the ESS and the overproduction mentioned previously.

In contrast to PV systems, the optimization results suggest that the installation of ST systems or wind turbines are not feasible under the given economic circumstances. Therefore, future changes in investment costs are likely to have a direct impact on the results of these optimizations. Hence, the ongoing price decrease, for example, for PV modules will further decrease the costs of the farm’s energy system. This is an important conclusion, as it allows the formulation of potential subsidies to accelerate the ongoing energy transition.

Furthermore, the obtained results indicate that the investment costs for ESS are only economically feasible if no grid connection is available (Scenario 2). Otherwise, the selling of surplus energy at a potentially low price yields increased revenues compared to an ESS investment. Thus, an ESS is only advantageous if the costs for procuring energy from the grid are high and for feeding into the grid low.

The optimization results show that the installation of a heating plant fueled by wood as the cheaper solution compared to a plant-oil-based CHP (see Scenarios 0 and 1). However, if autarky is the main goal (Scenario 2), the simultaneous production of heat and electricity by the CHP gains more importance and thus is one of the production plants in this scenario.

A sensitivity analysis indicates that changes in the electricity prices yield highly different results. For example, a 10 % decrease in the procurement price leads to mostly unchanged plant sizes, whereas a 10 % increase of the procurement price results in the maximum size of the PV system. The same effect can be observed if the feed-in price *c*_feed-in_ is increased by 50 %. Again, the maximum size of the PV system is chosen and thus the SCR sinks also to about 7 %. This is an important result, as it allows for the conclusion that the change in electricity prices, either for procurement or feed-in, has a major impact on the economical feasibility of RES-based systems. If more private citizens or entrepreneurs shall be motivated to invest into sustainable system a change in the available subsidy systems to increase feed-in tariff seems to be a highly promising approach.

One major drawback of the MILP optimization approach is that it relies heavily on the correctness of the provided input data. This concerns not only the price data (e.g., capital and operational expenditures) but also the underlying weather and demand data. Commonly, MILP problems rely on historical data and thus can only predict the future system behavior within certain limits. Therefore, it is important to continuously evaluate the input data and interpret the results with all potential uncertainties in mind. Additionally, it is almost impossible to predict the future development of energy and fuel prices, or investment costs for different production systems. Another aspect likely to affect the optimization results is the weather dependency of the RESs, particularly in the light of climate change. Despite these drawbacks, MILP optimization yield highly useful and very important results. For example, the fact that changes in electricity prices can on the one hand lead to a prominent rise in RESs investments and on the other hand decrease the annual costs of the resulting systems is a promising conclusion for the formulation of future subsidy systems or incentives.

Conclusion and Outlook

In this study a MILP formulation of an energy system of a typical Austrian farm based on RESs was presented. The optimization results indicate that the installation of RES-based production plants decreases the costs for the farm’s energy system. Furthermore, it was shown that full autarky for the investigated farm is possible if a CHP and an ESS are considered. A sensitivity analysis regarding the procurement and feed-in prices for electricity showed that subsidied feed-in tariffs are a highly effective tool to accelerate to building of renewable energy systems.

Future work will focus on improving the underlying price and weather data as well as on implementing forecasts of future price trends, e.g., for PV systems to decrease the uncertainties of the optimization results.

Availability of data and materials

This work is based on the Master thesis written by one of the authors, B.G., at the University of Applied Sciences Burgenland, Austria. The thesis includes additional data and optimization results.

Author’s contributions

B.G., I.V.T., and L.G. developed the MILP formulation of the energy system. B.G. implemented the model and conducted the optimizations. L.G. drafted most parts of the manuscript and supported the analysis of the results. C.P. gave substantial feedback on the data analysis and feedback on the manuscript. All authors have read and approved the manuscript.

Competing interests

The authors declare that they have no competing interests.

**Author details**

^1^ Fachhochschule Burgenland GmbH, Department of Energy and Environment, Steinamangerstraße 21, 7423, Pinkafeld, AT. ^2^ Forschung Burgenland GmbH, Campus 1, 7000, Eisenstadt, AT.

References

1. Tsai, S.-B., Xue, Y., Zhang, J., Chen, Q., Liu, Y., Zhou, J., Dong, W.: Models for forecasting growth trends in renewable energy. Renewable and Sustainable Energy Reviews 77, 1169–1178 (2017)

2. Statistik Austria: Gesamtenergiebilanz Osterreich 1970 bis 2018 - Detailinformation. http://www.statistik.at/web_de/statistiken/energie_umwelt_innovation_mobilitaet/energie_und_umwelt/energie/energiebilanzen/index.html

3. Machado, J.A.T., Ozedmir, N., Baleanu, D.: Mathematical Modelling and Optimization of Engineering Problems, 1st edn. Springer, Switzerland (2020)

4. Vielma, J.P.: Mixed integer linear programming formulation techniques. SIAM Review 57(1), 3–57 (2015)

5. Wang, Y., Zhang, N., Zhuo, Z., Kang, C., Kirschen, D.: Mixed-integer linear programming-based optimal configuration planning for energy hub: Starting from scratch. Applied Energy 210, 1141–1150 (2018)

6. Götze, J., Dancker, J., Wolter, M.: A general milp based optimization framework to design energy hubs. at Automatisierungstechnik 67(11), 958–971 (2019)

7. Scalfati, A., Iannuzzi, D., Fantauzzi, M., Roscia, M.: Optimal sizing of distributed energy resources in smart microgrids: A mixed integer linear programming formulation. In: 6th International Conference on Renewable Energy Research and Applications (ICRERA), pp. 568–573 (2017)

8. Nikkhah, A., Van Haute, S.: Energy flow modeling and optimization trends in food supply chain: a mini review. Current Opinion in Environmental Science & Health 13, 16–22 (2020)

9. Schmidt, J., Schönhart, M., Biberacher, M., Guggenberger, T., Hausl, S., Kalt, G., Leduc, S., Schardinger, I., Schmid, E.: Regional energy autarky: Potentials, costs and consequences for an austrian region. Energy Policy 47, 211–221 (2012)

10. Dawoud, S.M., Lin, X., Okba, M.I.: Hybrid renewable microgrid optimization techniques: A review. Renewable and Sustainable Energy Reviews 82, 2039–2052 (2018)

11. Loganathan, B., Chowdhury, H., Mustary, I., Rana, M.M., Alam, F.: Design of a micro wind turbine and its economic feasibility study for residential power generation in built-up areas. Energy Procedia 160, 812–819 (2019)

12. Austrian Power Clearing and Settlement Agency: Standard Load Profiles. https://www.apcs.at

13. Austrian Gas Clearing and Settlement Agency: Standard Load Profiles. https://www.agcs.at

## POSTER ABSTRACT

### P4 Quantification of Unidirectional Flexibility Potential of Battery Electric Vehicles using Real-world Mobility Data

#### Prakhar Mehta

##### School of Business, Economics and Society, FAU Erlangen-Nuremberg, Lange Gasse, 20, 90403, Nuremberg, Germany

###### **Correspondence**: Prakhar Mehta (prakhar.mehta@fau.de)

Summary

Battery Electric Vehicles (BEVs) are promising candidates to provide flexibility to the electric grid by adjusting their charging and discharging processes. However, the magnitude of their contribution, inclusive of human mobility behaviour, remains unclear. In this work, a real-world GPS-labelled mobility data set measured on 1000 cars across 2 years, comprising over 4 million trips, is used to simulate BEV charging and quantify the unidirectional flexibility potential provided by delaying charging. Flexibility is modelled as tuples of Power and Time for different durations at different times of the day. This work quantifies flexibility while accounting for real trips between home, work and public locations, and bears significance for grid operators to account for realistic flexibility potential in power system operation. Keywords: Unidirectional Flexibility; Battery Electric Vehicle; Mobility Behaviour

Introduction

Efforts to mitigate climate change have led to the adoption of intermittent renewable energy sources (RES) of electricity production and motivated the introduction of battery electric vehicles (BEVs) globally [1]. The existing electric grid infrastructure faces problems due to demand-supply imbalances which lead to overvoltages, frequency fluctuations and grid equipment overloading [2, 3]. Hence, the electric grid has an increased need for flexibility — that is, the ability to maintain stability during unforeseen changes in operational situations, which could be introduced from the demand-side, supply-side or other external factors [4, 5, 6]. While BEVs are primarily meant for mobility services, they lie idle 90-95% of the time daily [7]. Consequently, BEVs are inherent candidates to time-shift the electricity demand to times of oversupply [8]. Delaying the charging process of BEVs can make energy and power available instantaneously, termed as unidirectional flexibility, without the need for complex vehicle-to-grid (V2G) infrastructure. BEV batteries are hence an untapped flexibility resource, and it would be helpful to understand the realistic potential of this flexibility. Yet, existing studies rely on artificial or limited mobility profiles and do not fully address the uncertainty arising from human mobility behaviour, including charging behaviour, range anxiety, unforeseen trips, etc. Based on a set of high-resolution real-world mobility data, this work aims to answer the following overarching research question: *What is the unidirectional flexibility potential provided by a fleet of BEVs by delaying charging?*

Related Work

Prior research has investigated the flexibility potential arising from BEVs. Existing models quantify flexibility through optimal BEV charging, which helps increase RES utilization levels [9], also with the inclusion of user preferences [10]. Flexibility has also been quantified as effective SOC available for use as a distributed energy resource [11]. Optimizations provide best-case estimates of either upper or lower bounds, and are hence often unrealistic potentials. Further, quantification of flexibility as increasing RES utilization makes its direct quantification as a market product, for instance in electricity markets as balancing energy, difficult. [12] define flexibility from BEVs as a 3-dimensional tuple of time, power and energy flexibility, and quantify flexibility provided by 20,000 BEVs modeled through mobility surveys in Germany for each minute in August, ranging between 0-9 MW of positive (ramping down BEV load) and 100-150 MW of negative flexibility (ramping up BEV load). The studies described so far lack relevant real-world driving data, while a limited number of studies have either a small sample [13] or data from public charging stations [14] which may not be representative of true travel patterns.

Research Questions

This work aims to enhance existing flexibility estimates by employing real-world driving profiles, which implicitly incorporate human behaviour, to simulate a fleet of 1000 BEVs and report the range of flexibility over a year, in terms of Power for specified time slots. In order to highlight the impact of changing conditions and human behaviour, the flexibility potential is segmented based on the length of the *t*_*delay*_ command, the charging location of the BEVs, the driver types, and the BEV user range anxiety. The following research questions are addressed in this work:

1. RQ1: What is the unidirectional flexibility potential provided by a fleet of BEVs by delaying charging with a ‘*t*_*delay*_’ command, under the condition that the SOC level upon departure be the same as without the ‘*t*_*delay*_’ command?
  - What is the impact of the ‘*t*_*delay*_’ command duration, charger power, and location (Home, Work and Public) on the available flexibility potential?
  - How different is the flexibility potential provided by different BEV subfleets, based on driver types as classified in [15]?
2. RQ2: How does the calculated unidirectional flexibility change with future trip forecasts, and what trip forecast duration is relevant to calculate present flexibility?
3. RQ3: How does range anxiety among BEV users affect the flexibility potential?

Data and Methodology

The data set used in this work was collected by Octotelematics [16] between 2007 and 2009 in Northern Italy. It consists of 1000 internal combustion engine cars measured for location, speed, road-type and vehicle state aggregated for every 2 km driven. The data corresponds to over 4 million trips amounting to a distance of over 46.5 million km travelled [17]. Following the methodology employed in [17], the trip data is combined with high-resolution driving cycle speed profiles, assumed BEV parameters, and charging infrastructure to generate SOC profiles of the vehicles as BEVs. In order to not lose trip data, the vehicles are assumed to have a gasolinepowered range extender which only operates when the battery completely drains.

In this work, unidirectional flexibility is defined as power made available to the electric grid by temporarily delaying charging, and denoted as tuples of *Power* and *Time*: *F*_*t*_ =*< P,t >*. It is always positive, that is, V2G is not considered. One day (24 hours) is split into discrete time slots of length *t*_*delay*_. In each such time slot, the charging task (if the BEV is charging) is paused and only resumed in the future at the end of the time slot. That is, the charging task is effectively shifted *t*_*delay*_ minutes into the future by a ‘*t*_*delay*_ command’. The decision of whether a particular BEV can be shifted in this manner is based on set guidelines for the *SOC* required before the vehicle leaves for its next trip, varied across scenarios. Each vehicle can only be controlled by the *t*_*delay*_ command once in a day, to limit the duration of control of vehicles which can be inconvenient for the BEV user. Figure 1 shows how the *t*_*delay*_ command leads to shifting of the charging task into the future (Baseline Follower Scenario), and the consequent immediate availability of power (at the level of the charging power) which is quantified as the available unidirectional flexibility from that vehicle in that particular time slot (between 7 and 8 AM), given the mobility constraints it has. Considering *P*_*chg,t*_ to be the charger delivered power in kW and *x*_*i,t*_ a binary variable for each BEV *i* in time slot *t*, the aggregated unidirectional flexibility provided by a successful *t*_*delay*_ command across all *N* BEVs is given by equation 1. The resulting flexibility is reported as the range of power made available at every time slot *t* over a period of time - monthly, seasonally or even yearly.

1 $$ \underset{i=1}{\overset{N}{Fagg,t=\mathrm{X}\left( Pchg,i\times xi,t\right)}} $$

Three scenarios simulate conditions of different upfront knowledge of upcoming trips. In the Baseline Follower (BF) scenario addressing RQ1, flexibility is calculated under *t*_*delay*_ commands such that SOC levels are maintained the same as in the baseline (uncontrolled, charge as soon as possible scenario). The Upcoming Trip Needs (UTN) scenario addresses RQ2 and calculates flexibility with known knowledge of upcoming trips’ energy needs for varying future forecasts, and the Anxious Users scenario (AUS) incorporates BEV user range anxiety concerns through a model parameter of minimum SOC always requested.

Conclusion and Outlook

The methodology applied in this work aims to quantify the available unidirectional flexibility from a fleet of BEVs, based on real-world mobility data, thus accounting for human mobility behaviour. Results of the flexibility quantification based on the length of the *t*_*delay*_ command, driver type, charging location, future trip knowledge and BEV user range anxiety are expected to highlight patterns in order to access different amounts of flexibility under varying conditions across different times of day and the year. These potentials can help various stakeholders – energy aggregators, balancing groups and private individuals – access or provide flexibility according to their needs. Future research aims include BEV user preferences on charging frequency (daily/weekly) and trip uncertainty to improve calculated estimates.

Acknowledgements

The author would like to thank my primary advisors, Prof. Dr. Tiefenbeck and Prof. Dr. Staake, my shepherd Prof. Dr. Schumann, and the reviewers for their helpful comments and suggestions.

Funding

This work is funded by the Bavarian State Ministry of Science and the Arts, in a program coordinated by the Bavarian Research Institute for Digital Transformation (bidt) of the Bavarian Academy of Sciences, Germany.

Availability of data and materials

Not Applicable.

Competing interests

The author declares that they have no competing interests.

References

1. IEA . Global EV Outlook 2020. URL https://www.iea.org/reports/global-ev-outlook-2020 (2020)

2. Papadopoulos, P., Skarvelis-Kazakos, S., Grau, I., Cipcigan, L. & Jenkins, N. Electric vehicles’ impact on British distribution networks. *IET Electrical Systems In Transportation*. 2, 91-102 (2012,9)

3. Papaefthymiou, G. & Dragoon, K. Towards 100% renewable energy systems: Uncapping power system flexibility. *Energy Policy*. 92 pp. 69-82 (2016)

4. Cruz, M., Fitiwi, D., Santos, S. & Catalão, J. A comprehensive survey of flexibility options for supporting the low-carbon energy future. *Renewable And Sustainable Energy Reviews*. 97, 338-353 (2018)

5. Heffron, R., Körner, M., Shöpf, M., Wagner, J. & Weibelzahl, M. The role of flexibility in the light of the COVID-19 pandemic and beyond: Contributing to a sustainable and resilient energy future in Europe. *Renewable And Sustainable Energy Reviews*. 140 pp. 110743 (2021,4)

6. Andrey, C., Fournier, L., Gabay, M. & Sevin, H. The role and need of flexibility in 2030: focus on energy storage. *European Commission, Directorate-General For Energy*. pp. 83 (2019)

7. Bibak, B. & Tekiner-Moğulkoç¸, H. A comprehensive analysis of Vehicle to Grid (V2G) systems and scholarly literature on the application of such systems. *Renewable Energy Focus*. 36, 1-20 (2021)

8. Lund, P., Lindgren, J., Mikkola, J. & Salpakari, J. Review of energy system flexibility measures to enable high levels of variable renewable electricity. *Renewable And Sustainable Energy Reviews*. 45 pp. 785-807 (2015)

9. Schuller, A., Flath, C. & Gottwalt, S. Quantifying load flexibility of electric vehicles for renewable energy integration. *Applied Energy*. 151 pp. 335-344 (2015)

10. Sharifi, P., Banerjee, A. & Feizollahi, M. Leveraging owners’ flexibility in smart charge/discharge scheduling of electric vehicles to support renewable energy integration. *Computers And Industrial Engineering*. 149 (2020)

11. Mills, G. & MacGill, I. Assessing Electric Vehicle storage, flexibility, and Distributed Energy Resource potential. *Journal Of Energy Storage*. 17 pp. 357-366 (2018)

12. Schlund, J., Pruckner, M. & German, R. FlexAbility - Modeling and Maximizing the Bidirectional Flexibility Availability of Unidirectional Charging of Large Pools of Electric Vehicles. *E-Energy 2020 - Proceedings Of The 11th ACM International Conference On Future Energy Systems*, 121-132 (2020)

13. Zhao, H., Yan, X. & Ren, H. Quantifying flexibility of residential electric vehicle charging loads using non-intrusive load extracting algorithm in demand response. *Sustainable Cities And Society*. 50, 101664 (2019)

14. Sadeghianpourhamami, N., Refa, N., Strobbe, M. & Develder, C. Quantitive analysis of electric vehicle flexibility: A data-driven approach. *International Journal Of Electrical Power And Energy Systems*. 95 pp. 451-462 (2018)

15. Sodenkamp, M., Wenig, J., Thiesse, F. & Staake, T. Who can drive electric? Segmentation of car drivers based on longitudinal GPS travel data. *Energy Policy*. 130, 111-129 (2019)

16. Octotelematics. URL https://www.octotelematics.com/. Accessed 2021-07-02

17. Wenig, J., Sodenkamp, M. & Staake, T. Battery versus infrastructure: Tradeoffs between battery capacity and charging infrastructure for plug-in hybrid electric vehicles. *Applied Energy*. 255 pp. 113787 (2019,12)

**Fig. 1 (abstract P4).**
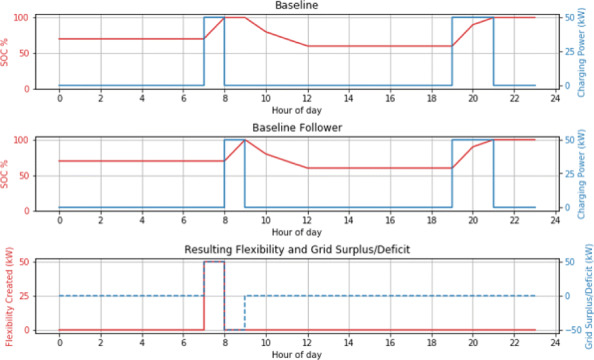
Flexibility provided by a BEV under a 1-hour *t*_*delay*_ command at 7 AM

## POSTER ABSTRACT

### P5 Balancing grid islands with distributed energy resources

#### Paul Hendrik Tiemann

##### Carl von Ossietzky University, Oldenburg, Ammerländer, Heerstraße 114-118, 26129 Oldenburg

###### **Correspondence:** Paul Hendrik Tiemann (paul.hendrik.tiemann@unioldenburg.de)

Summary

The European power system lacks a mechanism to allocate flexibility of distributed energy resources (DER) to operate distribution grids independently in case of a large area blackout. To level out grid island on a short time frame, reserves are needed which correspond to balancing services for frequency control. There is a research gap on how to allocate flexibility of DER for distribution grid islanding based on markets. This doctoral project sets out to develop a balancing market system in order to make frequency control flexibility accessible for transmission and distribution grid levels at the same time. In order to respect technical requirements as well as present-day regulation, an architecture on how to integrate a balancing market for distribution grids into the current market and control system in central Europe is developed. Furthermore, a game theoretical approach to develop and evaluate a suitable auction mechanism is presented. With the desired auction not only transmission grid operators, but also distribution grid operators shall be able to demand reserve amounts. The market operation is supposed to be compliant with the European regulation and objectives for balancing markets.

Keywords: Hierarchical auction; frequency control; island operation

Introduction

In case of a large area transmission grid blackout, the European electricity grid would broadly not be able to operate in grid islands without large conventional power plants. In order to increase the resiliency of the energy system, in a jointly edited statement from the project “Future Energy Systems”, renowned scientific academies postulate regulation for islanding capability by decentralized structures [1]. Distributed energy resources (DER) in large numbers could power a local electricity grid and stabilize grid islands to maintain a local energy supply if the transmission grid fails. Even though, the EU recently demanded grid operators to use predominantly market-based allocation processes in order to purchase non-frequency ancillary services [2, 3], there is no regulation for such operation yet.

Today in Europe, grid islanding is performed by large power stations. They are obliged to provide island operation capability [4, art 15(5), 16(1)]. However, this regulation does not apply to most distributed energy resources (DER) because of their small nominal power. If at least occasionally only few conventional generators are running, a coordination of DER will be needed in order to provide island operation capability without interruption. Therefore, a decoupled distribution grid as a microgrid in islanded mode [5] would need at least voltage and frequency control [6, p. 203], a protection architecture [6, p. 203] and resynchronization capability [5, p. 116] in order to operate independently from the transmission grid. This work focusses on frequency control. It raises the following research questions: *RQ:* What balancing market design could allocate flexibility for potential grid islands and is compatible with the harmonized European balancing system?

- *RQ-1:* What local market clearing should be performed with respect to game theory and a large number of DER?
- *RQ-2:* How to connect the result of the local market clearing and the LFC area-wide balancing market outcome?
- *RQ-3:* What game theoretic properties does the mechanism have to have with respect to the regulatory objectives for balancing markets?
- *RQ-4:* What bidding strategies does such a market design imply for local devices?

Method

In order to answer the research questions, on the one hand a market architecture is developed in order to allocate reserves for support of potential grid islands. On the other hand, a game theoretical model is set up to evaluate a proper market mechanism.

In order to be compatible with the European balancing system, the objectives of the EU for the LFC area-wide markets [3, art 3] are aspired. The present-day balancing system in Europe consists of an allocation and an activation system. If we keep the structure of the current transmission grid balancing system, a second market level would be needed. DSOs can then contract a sufficient amount of reserve to stabilize their grid in a possible islanding situation. This second market level will need its own market clearing procedure.

A first step at developing such a market system is to examine the allocation and activation mechanisms of the current balancing system as described above. The information flows, namely reserve offers, market result, control/activation signals, were analyzed in order to integrate a second market level. In case of FCR, the local activation by the controllers allows a lean market system without explicit activation paths. Balancing offers of DER can be aggregated at the distribution grid level and jointly sent to the LFC area market. After the market is cleared there, the results have to be transmitted back, so the second market level registers, which devices take part in balancing, and informs them. This way, it can also allocate additional capacity if needed. In figure 1, a possible market architecture for frequency containment reserve (FCR) is shown.

The development of an extension for existing balancing auctions lies in the area of market engineering [7]. Recent examples of an application in the energy system are presented in [8, 9], but do not respect balancing markets.

In this work, an auction for the second market level shall be developed to extend the load frequency control (LFC) area-wide balancing market. Here, mechanism design and auction theory shall be applied in order to respect the characteristics of the European balancing markets. Some relevant mathematical properties of mechanisms are economic efficiency, individual rationality, incentive compatibility and budget balance. However, it has been proven, that a mechanism can only have three of these properties at most if it implements a certain social choice function in dominant strategies. At the same time, the connected auction has to have basic functionality of an auction like a tie breaking mechanism [10, p. 336f]. It needs to be computational tractable [10, p. 274] in order to scale well with a high number of placed bids in order to integrate flexibility of small DER. The elements of the game theoretical analysis are summed up in figure 2.

Conclusion and Outlook

To level out power generation and consumption in grid islands on a short time frame, reserves are needed which correspond to balancing services for frequency control. There is a research gap on how to allocate frequency control flexibility of DER for distribution grid islanding based on markets. In order to respect technical requirements as well as present-day regulation, in this work, the current systems for frequency control and islanding operation were analyzed. An architecture on how to integrate a balancing market for distribution grids into the current market and control system in central Europe is developed. Furthermore, a game theoretical approach to develop and evaluate a designed auction mechanism is presented.

Next steps are, (1) a further refinement of the explicit steps of the necessary game theoretical evaluation, (2) extending an existing, prototypical action graph game to model the hierarchical auction in order to respect potential separations of distribution systems from the transmission grid.

Acknowledgements

This work is conducted at Carl von Ossietzky University Oldenburg under the supervision of Prof. Astrid Nieße. I would like to thank her for her advice and support. The prototypical software to solve the action graph game, which was hinted at, was implemented in cooperation with Gesa Ohlendorf whom I would like to thank as well.

Funding

This work is conducted within the research project ’SiNED – Systemdienstleistungen für sichere Stromnetze in Zeiten fortschreitender Energiewende und digitaler Transformation’ which is funded through the ’Niedersächsisches Vorab’ programme (grant ZN3563).

Availability of data and materials

No additional data or material has been used in order to conduct the presented work.

Author’s contributions

The literature research, the setup of assumptions, the development of the market architectures as well as the game theoretical approach were all performed by the author during his doctorate.

Competing interests

The author declares that he has no competing interests.

References

1. acatech – National Academy of Science and Engineerin, German National Academy of Sciences Leopoldina, Union of the German Academies of Sciences and Humanities: The Resilience of Digitalised Energy Systems Options for Reducing Blackout Risks (2021). Accessed: 2021-04-16. https://www.akademienunion.de/fileadmin/au-uploads/publikationen/Publikationen_PDFs/2021/ 2021_Position_Paper_ESYS_Digitalised_Energy_Systems.pdf

2. Parliament and Council of the European Union: Directive (EU) 2019/944 of the European Parliament and of the Council of 5 June 2019 on common rules for the internal market for electricity and amending Directive 2012/27/EU (2019). https://eur-lex.europa.eu/eli/dir/2019/944/oj

3. European Commission: Commission regulation (EU) 2017/2195 of 23 November 2017 establishing a guideline on electricity balancing (2017). https://eur-lex.europa.eu/eli/reg/2017/2195/oj

4. European Commission: Commission regulation (EU) 2016/631 of 14 April 2016 establishing a network code on requirements for grid connection of generators (2016). https://eur-lex.europa.eu/eli/reg/2016/631/oj

5. Farhangi, H., Joos, G.: Microgrid Planning and Design: A Concise Guide. Wiley-IEEE Press, Hoboken, New Jersey (2019)

6. Delfino, F., Procopio, R., Rossi, M., Brignone, M., Robba, M., Bracco, S.: Microgrid Design and Operation: Toward Smart Energy in Cities. Artech House power engineering series. Artech House and IEEE Xplore, Norwood, Massachusetts and [Piscataqay, New Jersey] (2018)

7. Weinhardt, C., Holtmann, C., Neumann, D.: Market-engineering. Wirtschaftsinformatik 45(6), 635–640 (2003). doi:10.1007/BF03250926

8. Mengelkamp, E., Notheisen, B., Beer, C., Dauer, D., Weinhardt, C.: A blockchain-based smart grid: towards sustainable local energy markets. Computer Science - Research and Development 33(1-2), 207–214 (2018). doi:10.1007/s00450-017-0360-9

9. Heilmann, E., Klempp, N., Wetzel, H.: Market Design of Regional Flexibility Markets: A Classification Metric for Flexibility Products and its Application to German Prototypical Flexibility Markets. https://www.uni-marburg.de/fb02/makro/forschung/magkspapers/paper_2020/02-2020_heilmann.pdf Accessed 03.04.2020

10. Shoham, Y., Leyton-Brown, K.: Multiagent Systems: Algorithmic, Game-theoretic, and Logical Foundations. Cambridge Univ. Press, Cambridge [u.a.] (2012)

**Fig. 1 (abstract P5).**
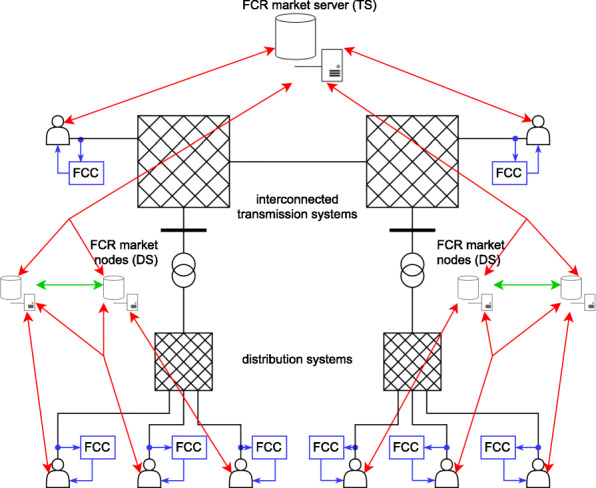
Exemplary architecture of a hierarchical balancing market for FCR with two market levels for the transmission system (TS) and distribution system (DS)

**Fig. 2 (abstract P5).**
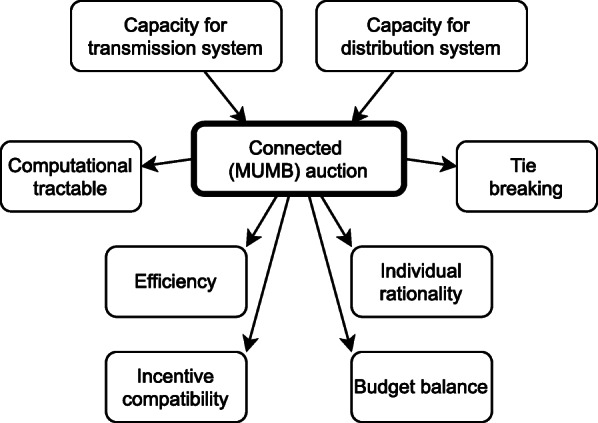
Drafted elements of the game theoretical analysis

## Footnotes

1 https://www.offis.de/en/offis/project/cybreslab.html

2 https://pandapower.readthedocs.io/en/v1.2.2/networks/cigre.html

